# Antimicrobial Peptide P‑113-DPS Suppresses the Cariogenic Virulence of Streptococcus mutans

**DOI:** 10.1021/acsabm.5c00314

**Published:** 2025-06-02

**Authors:** Qing Liu, Li Zhou, Simin Peng, Quan Li Li, Hai Ming Wong

**Affiliations:** † Paediatric Dentistry and Orthodontics, Faculty of Dentistry, 25809 The University of Hong Kong, Hong Kong SAR 999077, China; ‡ Institute of Oral Science, Department of Stomatology, Longgang Otorhinolaryngology Hospital, No. 3004L Longgang Avenue, Shenzhen 518172, China; § Key Lab of Oral Diseases Research of Anhui Province, College and Hospital of Stomatology, 12485 Anhui Medical University, Meishan Road, Hefei 230000, China

**Keywords:** antimicrobial peptide, Streptococcus mutans, cariogenic virulence, dental caries, dental biofilm

## Abstract

Dental caries is a widespread and contagious chronic infectious condition. As the principal cariogenic bacterium involved in dental caries, Streptococcus mutans (S. mutans) possesses cariogenicity-related properties, including acidogenicity, aciduricity, and exopolysaccharide (EPS) synthesis. Our previously designed peptide, P-113-DPS, has demonstrated antibacterial effects on S. mutans; however, its detailed impact on its cariogenic virulence factors remains unclear. This study focused on assessing changes in these factors following treatment with P-113-DPS. Furthermore, it aimed to investigate alterations in virulence-associated gene expression *in vitro*. The basic viability of S. mutans after P-113-DPS treatment was evaluated using a growth curve assay and 3-(4, 5-dimethylthiazol-2-yl)-2, 5-diphenyltetrazolium bromide staining assay. Acidogenicity was assessed through monitoring pH drop and lactate dehydrogenase activity, while aciduricity was evaluated through measuring survival rates in a lethal acidic environment. Additionally, EPS synthesis was analyzed using the anthrone sulfuric acid method, and structural observations were performed with confocal laser scanning and scanning electron microscopy. Finally, the changes in gene expression were examined utilizing quantitative real-time PCR (qPCR). P-113-DPS inhibited the growth and cell viability of S. mutans. Treatment with P-113-DPS resulted in decreased acidogenicity and aciduricity, evidenced by reduced acid production and survival rates at pH 5.0. Additionally, P-113-DPS suppressed the biofilm formation and EPS synthesis. Moreover, qPCR analysis revealed that P-113-DPS downregulated the expression of S. mutans virulence-associated genes. In conclusion, P-113-DPS exhibited strong antimicrobial properties and effectively suppressed the cariogenic virulence traits of S. mutans *in vitro* by downregulating virulence-associated genes, highlighting its promising anticaries potential.

## Graphical abstract

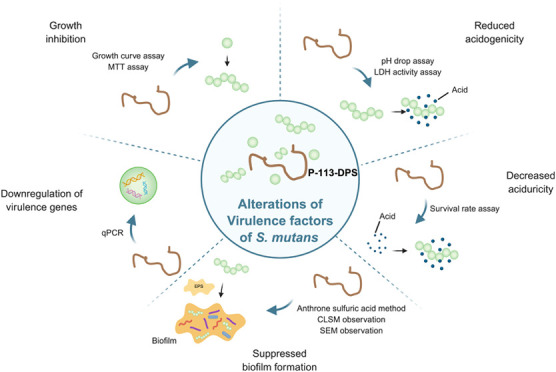

## 1 Introduction

Dental caries is a widespread and contagious chronic infectious condition, significantly affecting human health and quality of life.^1^ It is caused by cariogenic bacteria that metabolize free sugars in foods and drinks into acids, which gradually demineralize and destroy tooth structure over time.^2^ Streptococcus mutans (S. mutans), a key cariogenic bacterium in dental caries, possesses the cariogenicity-related properties, including acidogenicity, aciduricity, and exopolysaccharides (EPS) synthesis.^3,4^ S. mutans generates organic acids, primarily lactic acid, as a byproduct of carbohydrate metabolism, catalyzed by lactate dehydrogenase (LDH).^5,6^ These acids lead to a significant decrease in pH, serving as an important factor in the onset and development of dental caries. Furthermore, S. mutans can tolerate and survive in acidic environments (pH < 5.5) by activating the acid tolerance response, allowing it to maintain internal pH balance.^7^ This aciduricity ability enables S. mutans to dominate in the low-pH conditions of cariogenic biofilms, which further exacerbates the progression of dental caries.^8^ Additionally, S. mutans utilizes glucosyltransferases (GTFs) to produce both intracellular and extracellular polysaccharides from dietary sugars.^9^ Water-insoluble extracellular polysaccharides (EPS) serve as a structural framework for oral biofilms, facilitating the accommodation of various bacteria and promoting biofilm formation.^10^ Hence, suppressing the cariogenic virulence factors of S. mutans presents a promising strategy for dental caries prevention.

Notably, the virulence traits of S. mutans are regulated by an intricate network of systems, including two-component signal transduction systems, quorum sensing systems, acid tolerance responses, carbon catabolite pathways, DNA repair mechanisms, and the regulation of EPS synthesis.^11^ These systems enable *S. mutans* to adapt to environmental stresses such as acid pH, nutrient limitation, and competition with other microorganisms. Numerous studies have demonstrated that several genes within these regulatory systems, such as *gtfB*, *gtfC*, *gtfD*, *comD*, *comE*, *spaP*, and *gbpB*, are closely associated with key virulence traits of *S. mutans*, including acidogenicity, aciduricity, EPS synthesis, bacterial adhesion, and biofilm formation.^12,13^ Determining the alterations in the virulence-factor-related regulatory systems of S. mutans is crucial, which provides valuable insights into the management and prevention of dental caries.

Antimicrobial peptides (AMPs) have emerged as promising candidates for the treatment of infectious diseases, attracting considerable attention in recent years for their potential clinical applications.^14,15^ Compared to conventional antibiotics, AMPs exhibit potent antibacterial properties, lower risk of drug resistance, and enhanced drug loading and release capabilities.^16,17^ Recent advances in AMP synthesis have created new possibilities to prevent and manage dental caries.^18–21^ Our research team has developed an AMP P-113-DPS, which was shown to inhibit S. mutans adhesion and eradicate S. mutans biofilm on the tooth surface in our former study.^22^ However, its detailed roles in regulating the cariogenic virulence factors of *S. mutans* require further clarification.

The aim of this study was to evaluate the changes in cariogenic virulence traits of S. mutans *in vitro* following treatment with P-113-DPS, focusing on factors such as acid production, acid tolerance, EPS synthesis, biofilm formation, and biofilm architecture. Furthermore, alterations in virulence-related gene expression were analyzed. This study provides more comprehensive information on P-113-DPS, contributing to a better understanding of its anticaries potential.

## 2 Materials and Methods

### 2.1 Bacterial Cultivation

S. mutans UA159 was obtained from the Central Laboratory of the Faculty of Dentistry at the University of Hong Kong and cultured in brain–heart infusion (BHI) broth under anerobic conditions with 85% nitrogen, 10% hydrogen, and 5% carbon dioxide at 37 °C. After centrifuging at 5000 rpm for 10 min and washing using phosphate-buffered saline (PBS), the bacterial cells were resuspended in fresh BHI broth to achieve a final concentration of 1 × 10^6^ CFU/mL for subsequent experiments.

### 2.2 Peptide Synthesis

The antimicrobial peptide P-113-DPS (AKRHHGYKRKFH-S*p*S*p*) was synthesized, purified, and characterized following previously published methods.^22^ The peptide was kept at – 20 °C and prepared in 10 mM 4-(2-hydroxyethyl)-1-piperazineethanesulfonic acid (HEPES) buffer (pH = 7) for subsequent use. The minimal inhibitory concentration (MIC) of P-113-DPS against S. mutans was determined to be 32 μmol/mL. Sub-MICs concentrations (16, 8, and 4 μmol/mL) of P-113-DPS were used in this study.

### 2.3 Growth Curve Assay

Fresh S. mutans (1 × 10^6^ CFU/mL) was diluted 1:10 into BHI broth supplemented with P-113-DPS at sub-MIC levels (1/2, 1/4, and 1/8 MIC). BHI broth without P-113-DPS served as the control group, while BHI broth without bacteria served as the blank group. The growth of bacteria was tracked at 30 min intervals for 24 h using a microplate reader (CLARIOstar, BMG LABTECH, Germany) at 600 nm. The experiment was conducted independently on three separate occasions.

### 2.4 Cell Viability Assay

The viability of S. mutans cells after treatment with P-113-DPS at sub-MIC levels was assessed using 3-(4, 5-dimethylthiazol-2-yl)-2, 5-diphenyltetrazolium bromide (MTT) staining solution, prepared in PBS at a concentration of 0.5 mg/mL. Fresh S. mutans (1 × 10^6^ CFU/mL) was diluted 1:10 into BHI broth containing serial concentrations of P-113-DPS in a 96-well plate and incubated at 37 °C for 24 h under anerobic conditions. Postincubation, the bacterial suspensions were centrifuged (4500*g*, 5 min, 4 °C), with subsequent careful aspiration of the supernatants. A volume of 200 μL of MTT dye was then dispensed into each well, followed by a 2 h light-protected incubation at 37 °C. Following incubation, MTT solution was carefully aspirated and replaced with 200 μL of dimethyl sulfoxide (DMSO) to dissolve the resulting formazan crystals, followed by 20 min gentle shaking at ambient temperature. Then, aliquots (100 μL) were transferred to a new 96-well plate for optical density quantification at 540 nm. The viability percentages were calculated relative to those of the untreated controls. This experiment was repeated three times independently.

### 2.5 pH Drop Assay

The alterations in acid production of S. mutans following treatment with P-113-DPS at sub-MIC levels were quantified using a pH drop assay. S. mutans was inoculated into a BHI medium supplemented with 1% sucrose (BHIS broth) and grown under anerobic conditions at 37 °C for 24 h. Bacteria without P-113-DPS treatment served as the control group. pH levels in culture supernatants were determined using a pH meter (HI1131B, Hanna Instruments) at 0, 5, 10, and 24 h. The experiment was repeated independently three times.

### 2.6 LDH Activity Assay

S. mutans was diluted into BHIS broth containing serial concentrations of P-113-DPS, and then incubated anaerobically at 37 °C for 24 h. The LDH Activity Assay Kit (MAK066, Sigma-Aldrich) was utilized to perform the quantification of LDH activity. Following the manufacturer’s instructions, S. mutans cells were discarded via centrifugation (10,000*g*, 15 min, 4 °C). Supernatants were adjusted to 50 μL with the LDH Assay Buffer and transferred into a 96-well plate. Subsequently, a matching volume of the Master Reaction Mix was introduced into each well. Absorbance readings at baseline and end point were measured at 450 nm, and the absorbance difference (Δ*A*_450nm_) was derived. Results were calculated as the Δ*A*_450nm_ percentage relative to the untreated controls. This assay was independently repeated three times.

### 2.7 Acid Tolerance Assay

The role of different concentrations of P-113-DPS in modulating acid tolerance of S. mutans was evaluated through analyzing the bacterial survival following a 2 h exposure to pH 5.0. S. mutans was cultured to mid-logarithmic phase in tryptone yeast extract medium containing 20 mM glucose (TYEG) broth.^23^ Bacterial cells were then centrifuged and resuspended using TYEG broth supplemented with 40 mM phosphate/citrate buffer and graded P-113-DPS levels (pH 5.0). Before and after 2 h treatment, suspensions were diluted and spread onto the horse blood agar plates. After a two-day anaerobic incubation, colonies on the plates were counted. Acid tolerance was expressed as % survival rate: 100% × the colony number_(after treatment)_/ the colony number_(before treatment)_. The assay was independently performed in triplicate.

### 2.8 Biofilm Formation Assay

Crystal violet staining was employed to analyze the impact of different concentrations of P-113-DPS on S. mutans biofilm formation.^24^ Fresh S. mutans was diluted into BHIS broth containing serial concentrations of P-113-DPS and then incubated at 37 °C for 24 h under anaerobic conditions to facilitate biofilm development. The biofilm was rinsed twice using sterile PBS and treated with 200 μL 0.1% crystal violet solution for 15 min at room temperature. Three PBS wash cycles were employed to eliminate excess stain. Bound crystal violet was eluted utilizing 95% ethanol solution, and the SpectraMax iD5 plate reader (Molecular Devices, California) was employed to determine the optical density at 575 nm. This assay was independently performed three times.

### 2.9 Water-Insoluble EPS Measurement

Water-insoluble EPS levels in the S. mutans biofilm after P-113-DPS treatment were assessed through the anthrone method. After centrifugation (4,000 × g, 10 min, 4 °C), the formed biofilms were collected and resuspended in 200 μL of 0.4 mol/L NaOH, followed by incubation at 37 °C for 2 h. After a second centrifugation (6000 r/min, 10 min, 4 °C), the processed supernatant was combined with anthrone reagent in a 1:3 volumetric ratio and incubated at 95 °C for 8 min. A 100 μL aliquot was transferred to a fresh microplate for absorbance quantification at 625 nm. The experiments were independently performed three times.

### 2.10 Confocal Laser Scanning Microscope (CLSM) Observation of *S. mutans* Biofilm

*S. mutans* biofilms were constructed on tooth slices, which were prepared as described in our previous study,^22^ with ethical clearance provided by the Institutional Review Board of the University of Hong Kong/Hospital Authority Hong Kong West Cluster (IRB No. UW 24–562). Mature biofilms were stained using Alexa Fluor 647 (D22914; Molecular Probes) to label EPS and SYTO9 (S34854, Molecular Probes, Invitrogen, CA) to label bacteria according to the manufacturer’s guidelines. Bacterial cells emitted green fluorescence, while EPS exhibited red fluorescence. CLSM was used to observe the labeled biofilms, and ZEN 3.3 software (Carl Zeiss, Germany) was used to perform three-dimensional (3D) reconstructions. The bacteria and EPS biomasses were quantified using COMSTAT software.

### 2.11 Scanning Electron Microscope (SEM) Observation of S. mutans Biofilm

The S. mutans biofilms formed on tooth slices underwent two sterile PBS washes and were subsequently immobilized in 2.5% glutaraldehyde (Sigma-Aldrich) overnight at 4 °C. Processed biofilms were subjected to dehydration via serial ethanol solutions with increasing concentrations, each for 30 min, followed by critical point drying. Subsequently, the samples were sputter-coated and observed by using SEM (Hitachi, Hitachi High-Tech Corporation, Japan).

### 2.12 Virulence Gene Expression Profiling via Quantitative Real-Time PCR (qPCR)

To investigate the changes in expression of several virulence-related genes following P-113-DPS treatment, S. mutans were grown in BHI broth containing 1/2 MIC P-113-DPS for 1 h. Bacteria grown without the peptide served as the control group. RNA isolation was performed using the RNA-Quick Purification Kit (RN001, ES Science, China). cDNA synthesis was carried out by utilizing the HifairAdvanceFast One-step RT-gDNA Digestion SuperMix (11151ES60, Yeasen, China). Primer sequences for virulence-related genes (forward and reverse) are listed in Table 1. Each qPCR reactions were prepared with the synthesized cDNA and Hieff UNICONAdvanced qPCR SYBR Master Mix (11185ES08, Yeasen, China) following the manufacturer’s instructions. The qPCR was conducted utilizing the QuantStudio 6 Flex Real-Time System (Thermo Fisher Scientific), and fold changes in gene expression were determined by using the 2^–ΔΔCt^ method. All experiments were conducted independently in triplicate.

**Table 1:** Primer Sequence for Virulence-Related Genes

genes	primer sequence (forward and reverse)
16S rRNA	AGCGTTGTCCGGATTTATTG
	CTACGCATTTCACCGCTACA
*gtfB*	CACTATCGGCGGTTACGAAT
	CAATTTGGAGCAAGTCAGCA
*gtfC*	CCGACACCAACACCAGATCA
	TAAACAGGATCAGTCGGCGG
*gtfD*	TTGACGGTGTTCGTGTTGAT
	AAAGCGATAGGCGCAGTTTA
*spaP*	CCGACACCAACACCAGATCA
	TAAACAGGATCAGTCGGCGG
*gbpB*	AGCAACAGAAGCACAACCATCAG
	CCACCATTACCCCAGTAGTTTCC
*comE*	TTCCTCTGATTGACCATTCTTCTG
	GAGTTTATGCCCCTCACTTTTCAG
*ldh*	AAAAACCAGGCGAAACTCGC
	CTGAACGCGCATCAACATCA
*atpD*	TGTTGATGGTCTGGGTGAAA
	TTTGACGGTCTCCGATAACC
*wapA*	TGACTTTGACTGATGTTGTCGGAG
	GAAAAATCCTCAGCATAAGGTCGC

### 2.13 Statistics Analysis

Data analysis was conducted using SPSS 26.0 (IBM, New York). qPCR data were analyzed via *t*-test, while group comparisons for other experiments were performed via one-way ANOVA followed by Tukey’s posthoc test. Statistical significance was determined as *P* < 0.05.

## 3 Results

### 3.1 P-113-DPS Inhibited the Growth and Cell Viability of Planktonic *S. mutans*

Growth curve assay and MTT assay were performed to evaluate the growth and cell viability of S. mutans after P-113-DPS treatment, respectively. Treatment with P-113-DPS at the MIC concentration nearly completely inhibited bacterial growth and markedly reduced the cell viability of S. mutans (Supporting Information). Therefore, sub-MIC levels were used in subsequent experiments. Figure 1A demonstrated dose-dependent growth inhibition of S. mutans by P-113-DPS, manifesting as a delayed growth initiation and decreased final bacterial cell density compared to the control group. The MTT assay showed no significant bacterial viability change in the 1/8 MIC group compared to the untreated group. However, P-113-DPS at 1/4 MIC and 1/2 MIC resulted in a pronounced reduction in S. mutans viability (*P* < 0.001; Figure 1B).

**Figure 1:**
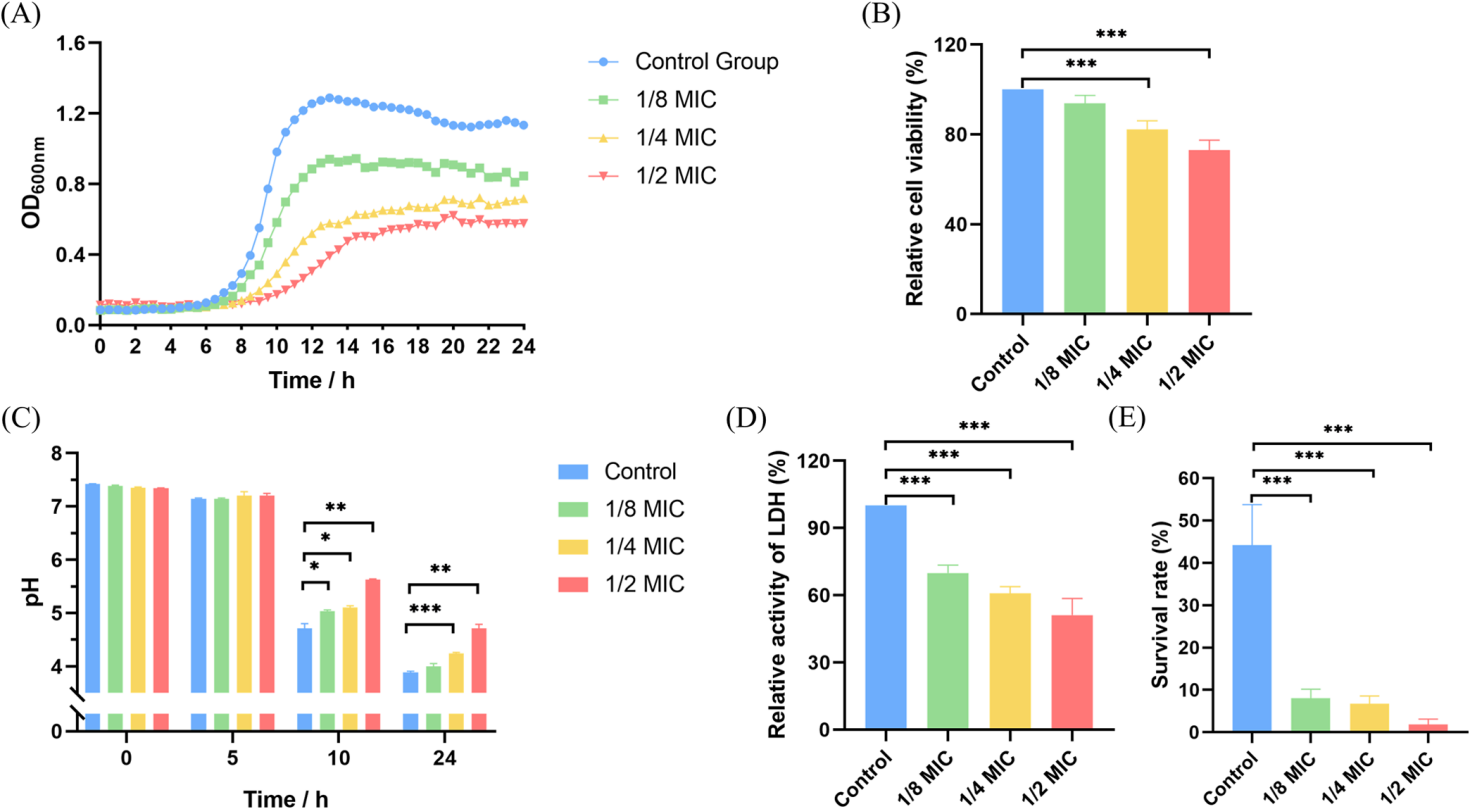
Effect of P-113-DPS at sub-MIC levels on the growth, cell viability, acidogenicity, and aciduricity of S. mutans. (A) Bacterial growth after P-113-DPS treatment was evaluated using a growth curve assay. (B) Relative cell viability of S. mutans after P-113-DPS treatment was assessed via MTT assay. (C) Acid production under P-113-DPS treatment was determined using the pH drop assay, and (D) LDH activity was measured to explore acidogenicity changes. (E) Acid tolerance following P-113-DPS treatment was evaluated by analyzing bacterial survival following a 2-h exposure to pH 5.0. (*P < 0.05, **P < 0.01, ***P < 0.001 compared to control group).

### 3.2 P-113-DPS Inhibited the Acidogenicity and Aciduricity of S. mutans

The pH drop assay and LDH activity assay were used to assess the changes in the acidogenicity of S. mutans following treatment with P-113-DPS at sub-MIC levels. As shown in Figure 1C, P-113-DPS at sub-MIC levels delayed the pH decrease after 10 h of treatment. After 24-h incubation, the 1/4 MIC and 1/2 MIC groups exhibited significantly higher final pH values compared to the untreated group (*P* < 0.001 and *P* < 0.01, respectively; Figure 1C). Additionally, LDH activity in S. mutans exhibited a concentration-dependent decline following treatment with P-113-DPS (*P* < 0.001, Figure 1D), demonstrating a reduction in the acidogenicity of *S. mutans*.

P-113-DPS also impaired the acid survival capability of S. mutans in a concentration-dependent manner. Exposure to lethal acidity (pH 5.0) induced a substantial reduction in the survival rate of S. mutans in the experimental group compared to the untreated control (*P* < 0.001, Figure 1E). Notably, P-113-DPS at 1/2 MIC suppressed the bacterial survival by nearly 99% compared to the untreated group (*P* < 0.001).

### 3.3 P-113-DPS Decreased the Biofilm Formation and EPS Synthesis

P-113-DPS treatment demonstrated significant inhibition of biofilm formation and EPS synthesis (Figure 2). The crystal violet staining assay revealed no statistically significant impact of P-113-DPS at 1/8 MIC on S. mutans biofilm formation compared to the control group. However, 1/4 MIC and 1/2 MIC treatments caused significant reductions in biofilm formation compared with the untreated control (*P* < 0.001, Figure 2A). Similarly, P-113-DPS at 1/8 MIC had no significant effect on the water-insoluble EPS synthesis in the biofilm. In contrast, EPS synthesis was significantly inhibited following treatment with P-113-DPS at 1/4 MIC and 1/2 MIC (*P* < 0.01 and *P* < 0.001, respectively; Figure 2B).

**Figure 2:**
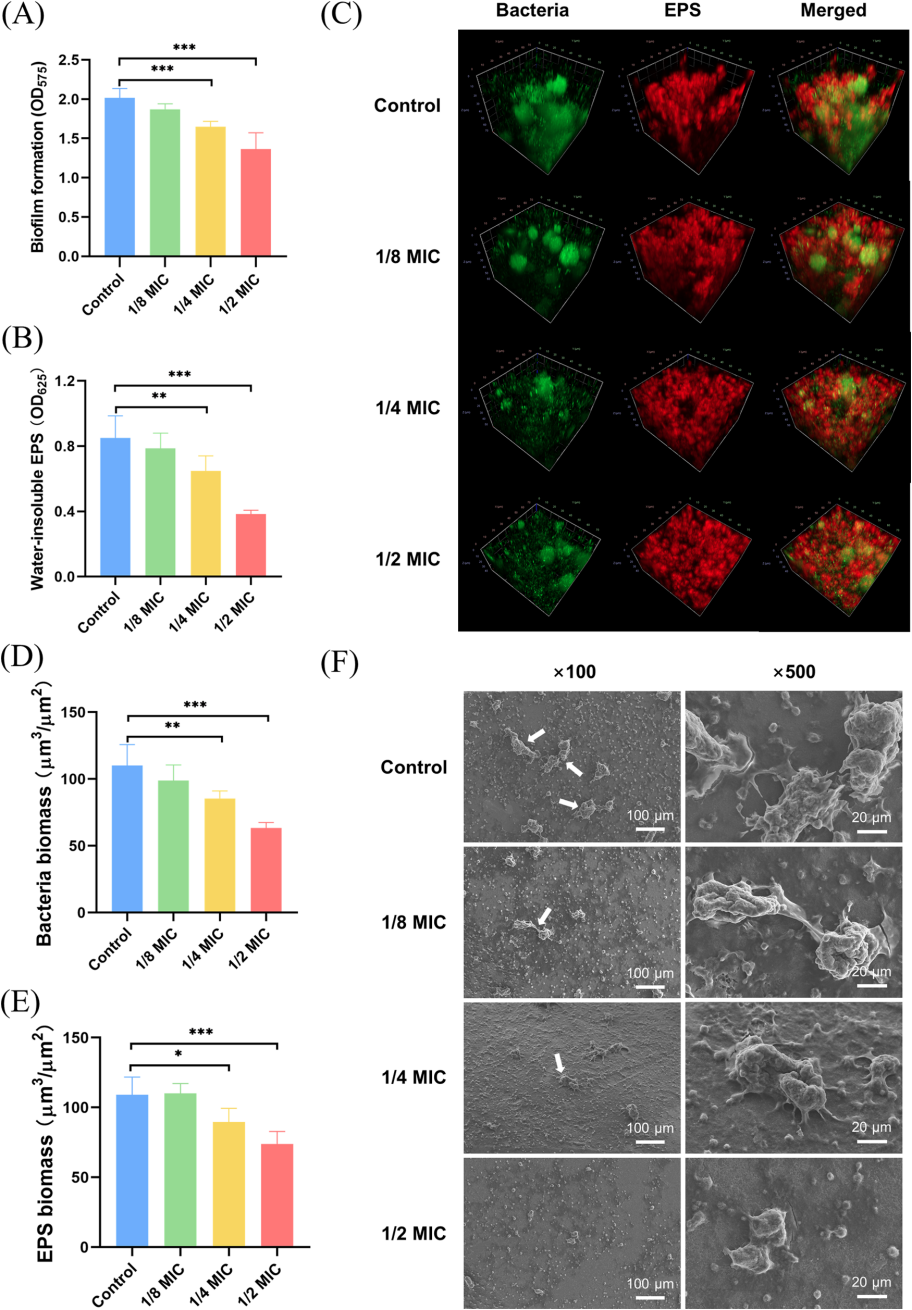
Effect of P-113-DPS at sub-MIC levels on S. mutans biofilm. (A) S. mutans biofilm formation following treatment with P-113-DPS was assessed using the crystal violet staining assay. (B) EPS synthesis in S. mutans biofilms after P-113-DPS treatment was assessed using the anthrone method. (C) Three-dimensional structure of the S. mutans biofilm following P-113-DPS treatment was visualized using CLSM, with bacterial cells stained green and EPS stained red. (D, E) Bacteria biomass (D) and EPS biomass (E) were quantified using COMSTAT software. (F) Morphology of the S. mutans biofilm following P-113-DPS treatment was observed using SEM. Arrows indicated the bacterial clusters consisting of cohesive S. mutans cells embedded in EPS. (*P < 0.05, **P < 0.01, ***P < 0.001 compared to the control group).

### 3.4 P-113-DPS Altered the Morphology of *S. mutans* Biofilm

CLSM and SEM observations were performed to assess the morphological alterations of the S. mutans biofilm following P-113-DPS treatment. The S. mutans biofilm in the control group displayed robust EPS accumulation (red fluorescence) and abundant bacterial colonization (green fluorescence, Figure 2C). Treatment with P-113-DPS at 1/8 MIC did not result in noticeable changes in EPS or bacterial distribution. Biofilms treated with P-113-DPS at 1/4 MIC and 1/2 MIC appeared thinner and looser. Additionally, quantitative fluorescence analysis demonstrated that P-113-DPS at 1/4 MIC and 1/2 MIC significantly reduced both EPS and bacterial biomass compared with the control group (*P* < 0.05, Figure 2D,E). SEM observations further confirmed a reduction in biofilm formation following P-113-DPS treatment (Figure 2F). The S. mutans biofilm treated with 1/8 MIC of P-113-DPS showed no noticeable morphological differences from the untreated control, displaying cohesive S. mutans cells embedded in dense extracellular matrices. The matrix density decreased along with reduced bacterial colonization in the S. mutans biofilm following treatment with 1/4 MIC of P-113-DPS. Moreover, treatment with 1/2 MIC of P-113-DPS significantly inhibited biofilm formation, resulting in only scattered bacterial adhesion (Figure 2F).

### 3.5 P-113-DPS Downregulated the Virulence-Related Gene Expression

Based on the above results, 1/2 MIC of P-113-DPS substantially inhibited the virulence traits of S. mutans. Therefore, we selected the 1/2 MIC concentration of P-113-DPS to further investigate alterations in gene expression following the treatment. The expression of virulence-related genes in S. mutans after treatment with 1/2 MIC of P-113-DPS is shown in Figure 3. P-113-DPS at 1/2 MIC significantly downregulated the expression of virulence-associated genes, including *gtfB*, *gtfC*, *gtfD*, *spaP*, *gbpB*, *comE*, *ldh*, *atpD*, and *wapA* (*P* < 0.05, Figure 3).

**Figure 3:**
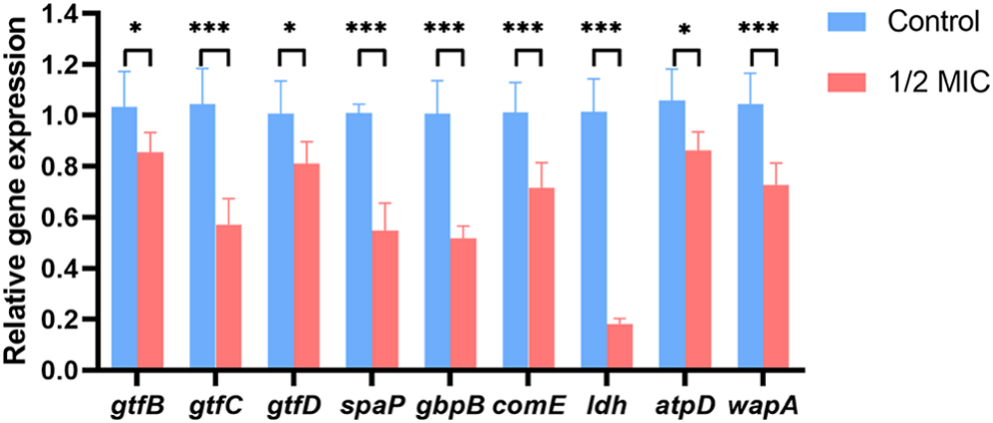
Cariogenic virulence-related gene expression alterations in S. mutans following P-113-DPS treatment. The expression of several cariogenicity-related genes in S. mutans treated with P-113-DPS at 1/2 MIC was evaluated using the qPCR method. 16S rRNA was utilized as an internal control for normalizing virulence-associated gene expression. (*P < 0.05, **P < 0.01, ***P < 0.001 compared with the untreated control).

## 4 Discussion

In our previous study, we developed the peptide P-113-DPS, which was shown to inhibit S. mutans adhesion and eradicate S. mutans biofilm on the tooth surface.^22^ To further explore the antibacterial mechanisms of P-113-DPS, we evaluated its impacts on the cariogenic virulence traits of S. mutans. Our findings demonstrated the suppressed cariogenic virulence of S. mutans following P-113-DPS treatment, with decreased acidogenicity, aciduricity, biofilm formation, and EPS synthesis, highlighting its promising anticaries potential as an anticaries agent.

The MIC of P-113-DPS against S. mutans UA159 was determined to be 32 μmol/mL. The MIC was determined using a slightly modified experimental setup compared with our previous study, with variations in bacterial strain, culture conditions, and assay protocols, which may have contributed to the higher MIC value. However, P-113-DPS treatment at the MIC level is not suitable for assessing the alterations in cariogenic virulence of S. mutans, as it nearly completely inhibits the visible bacterial growth. Therefore, sub-MIC concentrations of P-113-DPS were selected for further investigation. Cariogenic virulence factors of S. mutans enable it to colonize tooth surfaces, form plaque biofilms, produce acid, and survive in acidic conditions, establishing it as a primary contributor to dental caries.^3^ First, the basic viability of S. mutans allows it to survive and maintain metabolic activity under diverse environmental conditions. The results of the growth curve assay and MTT staining assay showed that treatment with P-113-DPS at 1/4 and 1/2 MIC levels significantly inhibited S. mutans viability, which may induce a reduced ability to proliferate and adapt to environmental stress, ultimately diminishing its pathogenicity and weakening its cariogenic potential.

As the principal cariogenic bacterium, one of the key traits of S. mutans is its acidogenicity.^8^ Treatment with P-113-DPS delayed the pH decrease and suppressed LDH activity, indicating an impairment in the acidogenicity of S. mutans. During carbohydrate fermentation, S. mutans generates organic acids (primarily lactic acid), which lower the external environmental pH levels and initiate enamel demineralization.^25^ LDH is the vital enzyme in the metabolic process of S. mutans and is essential for its acidogenicity.^26^ Our findings revealed that the expression of the *ldh* gene in S. mutans was reduced following exposure to P-113-DPS at 1/2 MIC. Consequently, inhibition of LDH activity can significantly impair the acidogenic potential of S. mutans, thereby weakening its ability to contribute to the development of carious lesions.

Another key trait of S. mutans is its aciduricity, which enables it to survive and grow in acidic environments.^7^ Furthermore, the F_1_F_0_-ATPase, a membrane-bound enzyme complex, serve as the primary mechanism for proton transport across the S. mutans cell membrane, maintaining pH homeostasis and playing a vital role in its acid tolerance.^27^ Our results showed that P-113-DPS treatment decreased the survival rate of S. mutans under lethal conditions and downregulated gene *atpD* (encoding F_1_F_0_-ATPase) expression, thereby impairing its acid resistance ability.

S. mutans is pivotal in forming architecturally complex biofilm on tooth enamel.^28^ Through adhering to the tooth surface and predominating in the dental biofilm, S. mutans initiates tooth demineralization and promotes caries progression.^3^ S. mutans utilizes the GTFs system to metabolize dietary sucrose and synthesize glucans, including EPS. These glucans strengthen S. mutans adhesion to tooth enamel and promote microbial coaggregation, thereby thickening the biofilm and enhancing its resistance to host clearance mechanisms and antimicrobial agents.^28,29^ The interaction between salivary agglutinins and *spaP*-encoding protein (also known as the I/II antigen) is critical for S. mutans colonization and biofilm formation.^30^ P-113-DPS significantly inhibited biofilm formation and the synthesis of water-insoluble EPS at higher concentrations (>1/8MIC). Furthermore, down-regulated expression of *gtfB*, *gtfC*, *gtfD*, and *spaP* in S. mutans was observed following treatment. Morphological observations further confirmed the reduced bacterial cells and extracellular matrix, indicating an impaired ability for biofilm formation and EPS synthesis.

Considering that 1/2 MIC of P-113-DPS significantly suppressed the virulence factors of *S. mutans*, we subsequently examined the expression changes in several key genes involved in regulatory systems that enable *S. mutans* to adapt to environmental stress. In this study, P-113-DPS at 1/2 MIC downregulated the expression of *gtfB*, *gtfC*, and *gtfD*, which are critical for glucan matrix production.^31^ These glucans enable S. mutans to adhere tightly to the tooth surface and are closely associated with biofilm formation.^32^ Additionally, the expression of *spaP* and *gbpB* was reduced following treatment, and these genes are known to mediate bacterial attachment and contribute to plaque formation.^33,34^ The inhibition of adhesion-related and glucan-synthesis-related genes resulted in decreased EPS production and biofilm formation. comE is a component of the quorum-sensing cascade in S. mutans and plays a role in the two-component signal transduction system, which mediates bacterial adaptation to environmental stress and modulates multiple virulence traits.^12^ The expression of *ldh* (encoding LDH) was also decreased following treatment, potentially accounting for P-113-DPS-mediated inhibition of the acidogenicity of S. mutans. Moreover, the reduced aciduricity may result from the downregulated expression of *atpD* in S. mutans, which encodes the proton pump F_1_F_0_-ATPase. This enzyme facilitates H^+^ ions efflux from bacterial cells, helping S. mutans endure acid stress and maintain acid tolerance.^35^ Notably, the cell wall-related gene *wapA* was also downregulated after treatment, which may affect cell surface structure and biofilm formation.^36^ Overall, as shown in Figure 4, P-113-DPS treatment suppressed the virulence factors of S. mutans through downregulating several virulence genes, highlighting its promising anticaries potential.

**Figure 4:**
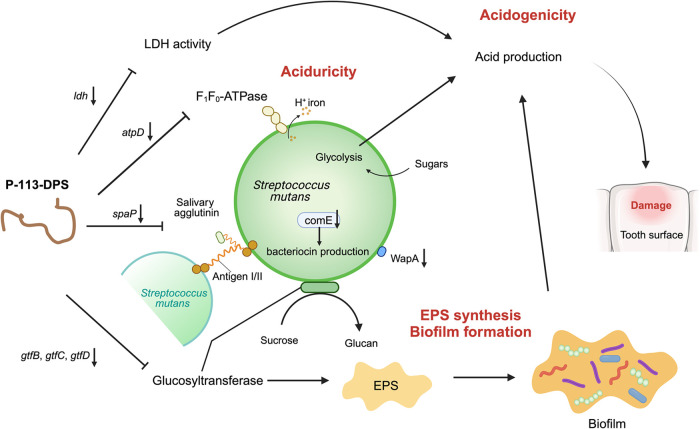
Diagram illustrating the suppressed virulence factors of S. mutans mediated by P-113-DPS. Created with BioRender.com.

The current study primarily focuses on the alterations in S. mutans virulence following treatment with P-113-DPS *in vitro*, highlighting its potential as a promising anticaries agent for caries prevention. However, the development of dental biofilms involves multiple microorganisms. Therefore, further research is needed to evaluate the effects of P-113-DPS on multispecies biofilms. It is also worth noting that the antifungal activity of peptide P-113 has been previously reported,^37^ suggesting that P-113-DPS may also possess antifungal properties, which warrants further investigation. Additionally, while this study provides evidence of P-113-DPS’s regulation of several cariogenicity-related genes, further analyses (such as transcriptomics, proteomics, and metabolomics) are required to better understand its mechanisms of action. Finally, the effectiveness and biocompatibility of P-113-DPS should be evaluated *in vivo* and through clinical applications to fully assess its potential for practical use.

## 5 Conclusions

In conclusion, P-113-DPS impaired the cariogenic virulence factors of S. mutans by suppressing its growth, reducing its acidogenicity and aciduricity, and downregulating the expression of virulence-associated genes. These effects highlight the potential of P-113-DPS as a promising candidate for clinical application in caries prevention.

## Supplementary Material



## Notes

Growth and cell viability of S. mutans following P-113-DPS treatment at MIC and sub-MIC levels (PDF)
