# Supplementary material for: Antimicrobial Peptide P‑113-DPS Suppresses the Cariogenic Virulence of Streptococcus mutans
Source: ACS Appl Bio Mater. 2025 Jun 2;8(6):4973–80. doi: 10.1021/acsabm.5c00314 (PMC12175158; doi:10.1021/acsabm.5c00314)
Supplement: Supplementary file 1 [file mt5c00314_si_001.pdf]

Supporting Information

Antimicrobial Peptide P-113-DPS Suppresses the Cariogenic Virulence of *Streptococcus mutans*

Qing Liu [a], Li Zhou [a], Simin Peng [a], Quan Li Li [b,c], and Hai Ming Wong [a]*

[a] Paediatric Dentistry and Orthodontics, Faculty of Dentistry, The University of Hong Kong, Hong Kong SAR 999077, China

[b] Institute of Oral Science, Department of Stomatology, Longgang Otorhinolaryngology Hospital, No. 3004L Longgang Avenue, Shenzhen, 518172, China

[c] Key Lab of Oral Diseases Research of Anhui Province, College and Hospital of Stomatology, Anhui Medical University, Meishan Road, Hefei, 230000, China

Corresponding author email: wonghmg@hku.hk

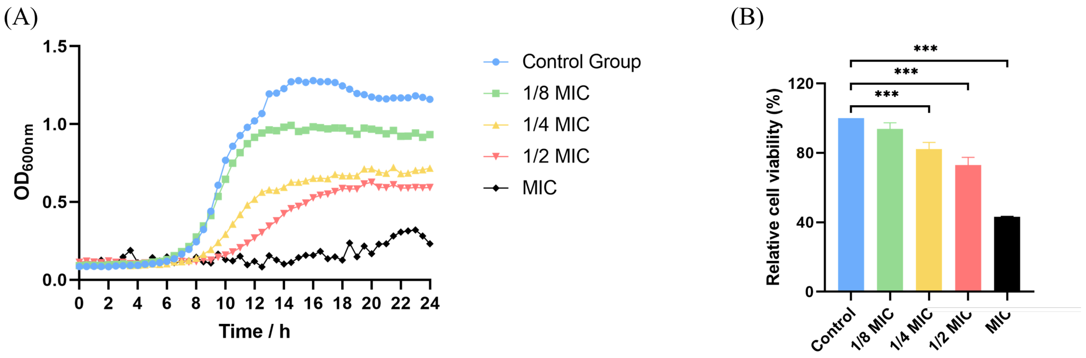

Suppplementary Figure. Effect of P-113-DPS at MIC and sub-MIC levels on the growth and cell viability of planktonic *S. mutans*. (A) Bacterial growth after P-113-DPS treatment was evaluated using growth curve assay. (B) The relative cell viability of *S. mutans* after P-113-DPS treatment was assessed via MTT assay.
